# Liver dysfunction in adults with COVID‐19 infection: A longitudinal study with transient elastography evaluation

**DOI:** 10.1002/jgh3.13118

**Published:** 2024-08-07

**Authors:** Ruveena Bhavani Rajaram, Thevaraajan Jayaraman, Xin‐Hui Khoo, Nalliah Saravanaa, Anjanna Kukreja, Bushra Megat Johari, Nadia Fareeda Muhammad Gowdh, Wai‐Kin Lee, Choong‐Yeong Sooi, Sazali Basri, Rong‐Xiang Ng, Hang‐Cheng Ong, Pui‐Li Wong, Sharifah Faridah Syed Omar, Sanjiv Mahadeva

**Affiliations:** ^1^ Gastroenterology Unit, Medical Department Universiti Malaya Medical Centre Kuala Lumpur Malaysia; ^2^ Gastroenterology Unit, Department of Medicine, Faculty of Medicine Universiti Teknologi MARA Sungai Buloh Malaysia; ^3^ Infectious Disease Unit, Medical Department Universiti Malaya Medical Centre Kuala Lumpur Malaysia; ^4^ Radiology Department Universiti Malaya Medical Centre Kuala Lumpur Malaysia; ^5^ Medical Department Hospital Seberang Jaya Seberang Jaya Malaysia; ^6^ Medical Department Hospital Tengku Ampuan Afzan Kuantan Malaysia

**Keywords:** chronic liver disease, COVID‐19 infection, liver dysfunction, liver injury, metabolic‐associated fatty liver disease

## Abstract

**Background and Aim:**

Abnormal liver biochemistry (ALB) is common among patients with COVID‐19 infection due to various factors. It is uncertain if it persists after the acute infection. We aimed to investigate this.

**Methods:**

A multicenter study of adult patients hospitalized for COVID‐19 infection, with at least a single abnormal liver function test, was conducted. Detailed laboratory and imaging tests, including transabdominal ultrasound and FibroScan, were performed at assessment and at 6‐month follow‐up after hospital discharge.

**Results:**

From an initial cohort of 1246 patients who were hospitalized, 731 (58.7%) had ALB. A total of 174/731 patients fulfilled the inclusion criteria with the following characteristics: 48.9% patients had severe COVID‐19; 62.1% had chronic liver disease (CLD); and 56.9% had metabolic‐associated fatty liver disease (MAFLD). ALB was predominantly of a mixed pattern (67.8%). Among those (55.2%) who had liver injury (aspartate aminotransferase/alanine aminotransferase >3 times the upper limit of normal, or alkaline phosphatase/γ‐glutamyl transferase/bilirubin >2 times the upper limit of normal), a mixed pattern was similarly predominant. Approximately 52.3% had normalization of the liver lunction test in the 6‐month period post discharge. Patients with persistent ALB had significantly higher mean body mass index (BMI) and serum low‐density lipoprotein (LDL), higher rates of MAFLD and CLD, higher mean liver stiffness measurement and continuous attenuated parameter score on FibroScan, and higher rates of liver injury on univariate analysis. Multivariate analysis was not statistically significant.

**Conclusions:**

Approximately 47.7% of COVID‐19 patients were found to have persistent ALB up to 6 months following the acute infection, and it was associated with raised BMI, elevated serum LDL, increased rates of MAFLD and CLD, and higher rates of liver injury on univariate analysis, but not on multivariate analysis.

## Graphical abstract

In a longitudinal study of 174 adult COVID‐19 patients with abnormal liver function, 52.3% achieved normalization of LFT at 6 months post COVID‐19 infection. With additional ultrasound imaging and Transient Elastography evaluation, COVID‐19 patients with a higher BMI, serum LDL, LSM, CAP score, underlying MAFLD and with moderate/severe COVID‐19 infection were more likely to have persistent liver dysfunction beyond 6 months.

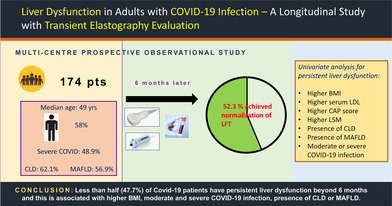

## Introduction

Since the emergence of coronavirus disease 2019 (COVID‐19), which caused a novel severe acute respiratory syndrome coronavirus 2 (SARS‐CoV‐2) in December 2019, over 757 million confirmed cases and over 6.8 million deaths have been reported globally.^1^ Variants of concern have been evolving since the beginning of the pandemic, and vaccines have been largely effective in containing the spread of the virus in the general population.^2^ Abnormal liver biochemistry (ALB) in COVID‐19 are common, and its prevalence was reported to be between 19% and 67%.^3^, ^4^, ^5^ Patients with severe COVID‐19 infection were found to have significantly higher pooled mean AST (aspartate aminotransferase) and ALT (alanine aminotransferase) levels, with the mean AST level being higher than the mean ALT level.^6^ Prevalence of chronic liver disease (CLD) among patients with COVID‐19 was around 3–4% and was associated with more severe infection and increased mortality.^7^, ^8^, ^9^, ^10^

Earlier reports indicated that liver impairment in COVID‐19 “may not have serious clinical consequences”.^11^ However, recent evidence has found that the presence of liver injury was associated with elevated pro‐inflammatory cytokines and was an independent prognostic factor for COVID‐19.^12^ There are multiple underlying mechanisms to explain liver function impairment, such as drug‐induced liver injury, viral‐induced cytopathic effect, exacerbation of a pre‐existing liver condition, and hypoxic liver injury.^13^ Numerous studies have been reported on liver dysfunction in COVID‐19, which were mostly retrospective and cross‐sectional in nature. These study designs were not able to explore whether the liver dysfunction persisted post COVID‐19 or whether there were long‐term sequelae.

In the present study, we investigated patients with COVID‐19 and ALB prospectively, and followed them up for a period of not exceeding 6 months. Additionally, we also explored the role of transient elastography as a predictor of persistent ALB.

## Methods

### Study design and participants

This study was conducted in accordance with the principles of the Declaration of Helsinki and approved by the University Malaya Medical Centre Research Ethics Committee (MECID No. 202146‐10 036). We performed an observational cohort study and recruited patients using a convenience sampling method. Patients aged 18 years and older who were admitted to Universiti Malaya Medical Centre and Hospital UiTM (both tertiary academic medical centers which were involved in the management of COVID‐19 patients during the pandemic) were invited to participate in the study provided they satisfied the following inclusion criteria:

1. Laboratory‐confirmed COVID‐19 based on real‐time reverse‐transcriptase polymerase‐chain‐reaction (RT‐PCR) assay for nasal and pharyngeal swab specimens.
2. Had at least a single abnormal liver function test (LFT).

Patients were recruited into the study after obtaining informed consent. All study patients were then subjected to blood investigations such as viral hepatitis B and C and autoimmune serology to exclude common causes of CLD. All patients were then invited for a review in the outpatient clinic with repeat LFT 4–6 months after discharge, after undergoing transient elastography and ultrasound imaging of the abdomen at the same time. In this manner, the liver stiffness measurement (LSM) readings would not be affected in patients with persistent mild elevation of liver enzymes or those with complete resolution.

### Data collection

Baseline clinical, laboratory, and anthropometric data were obtained by reviewing the electronic medical records of the patients. Prevalence of abnormal LFT were documented and categorized into hepatocellular injury (ALT or AST >40 U/L), cholestatic injury [alkaline phosphatase (ALP) >130 U/L or γ‐glutamyl transferase (GGT) >50 U/L], or mixed type (if both patterns of injury were present). Additionally, if AST or ALT was >3 times the upper limit of the normal value, or ALP/GGT/total bilirubin was >2 times the upper limit of their normal value, the patient was categorized as having liver injury. Clinical information, laboratory tests, and imaging results regarding current infection, pharmacotherapy prior to and during hospitalization for COVID‐19 infection, disease severity, use of mechanical ventilation, and specific outcomes were documented. Information regarding the presence of liver disease diagnosed either prior to or during admission in addition to presence of any clinical, biochemical, and features of liver dysfunction (i.e., presence of hepatic encephalopathy, ascites, jaundice, prolonged INR/PT) were also documented. During their follow‐up visit, the repeated liver biochemistry results were recorded. The results of liver ultrasound, specifically on the presence of hepatic steatosis, liver cirrhosis, or any other liver abnormality, as well as the liver stiffness measurement (LSM) and continuous attenuated parameter (CAP) value from FibroScan were documented.

### Sample size

Based on the proportion of 73.4% of normalized transaminases 6 months post COVID‐19 according to previous research,^14^ with 90% confidence interval and a margin of error of 5%, a minimum sample size of at least 182 patients was required.

### Statistical analysis

Categorical variables are presented as numbers and percentages. Chi‐square tests and Fisher's exact tests were used for categorical variables. Continuous values were expressed as means (standard deviations) and were calculated using the Student *t*‐test or one‐way ANOVA for parametric data. Continuous values were expressed as median [interquartile range (IQR)] and were calculated using the Mann–Whitney U or Kruskal–Wallis H test for nonparametric data. All statistical analyses were performed using SPSS (Statistical Package for the Social Sciences) Version 26.0. A value of *P* <0.05 is considered as statistically significant. Parameters with *P* <0.2 in univariate analysis were included for multivariate analysis.

## Results

**Figure 1:**
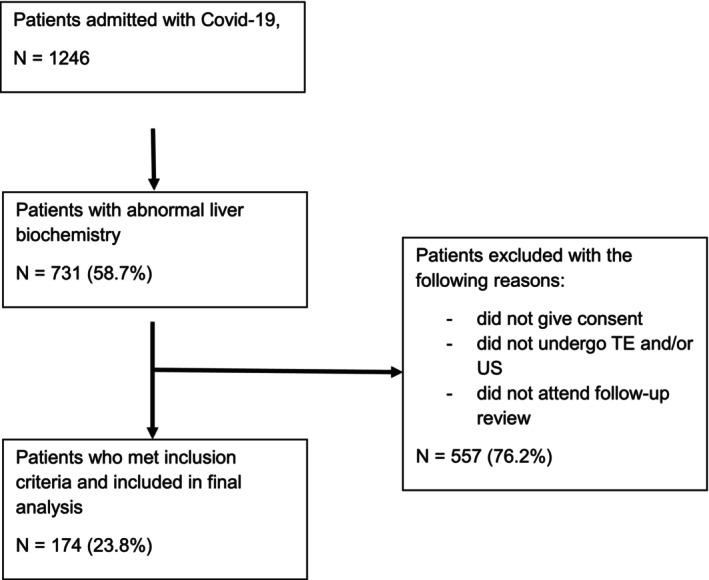
Flowchart of participant recruitment into the study.

From an initial cohort of 1246 patients who were hospitalized, 731 (58.7%) had ALB and 174 patients fulfilled the inclusion criteria and were included in the final analysis (Fig. 1). Table 1 outlines the demographic and baseline characteristics of the patients. The median (IQR) age was 49 (36–60) years, and a vast majority (75.9%) were below the age of 60 years. There was a preponderance of male gender (58%), and the ethnic distribution was as follows: 58.6% Malay, 21.3% Chinese, 17.2% Indian, and 2.9% others.

**Table 1:** Comparison of demographics and clinical characteristics between COVID‐19 patients with abnormal liver biochemistry (*n* = 174) and the baseline cohort (*n* = 1246)

Characteristics		*n* = 174	*n* = 1246	*P*‐value
Age	Median (IQR), years	49 (36–60)	50.0 (31–59)	
	1–60 years, *n* (%)	132 (75.9)	846 (67.9)	0.03
	>60 years, *n* (%)	42 (24.1)	400 (32.1)	
BMI, kg/m^2^ [Median (IQR)]		29.5 (26.25–33.78)	—	
Gender, *n* (%)	Male	101 (58.0)	618 (49.6)	0.04
	Female	73 (42.0)	628 (50.4)	
Ethnicity, *n* (%)	Malay	102 (58.6)	779 (62.5)	
	Chinese	37 (21.3)	233 (18.7)	0.36
	Indian	30 (17.2)	189 (15.2)	0.39
	Others	5 (2.9)	45 (3.6)	0.77
COVID‐19 severity, *n* (%)	Mild (CAT 1 & 2)	54 (31.0)	649 (52.1)	
	Moderate (CAT 3)	35 (20.1)	200 (16.1)	0.002
	Severe (CAT 4 & 5)	85 (48.9)	397 (31.9)	<0.001
Delta variant, *n* (%)	Yes	100 (57.5)	549 (44.1)	<0.001
	No	74 (42.5)	697 (55.9)	
Median duration of illness on admission, (IQR), days		6 (4–9)	—	
Length of hospitalization, median (IQR), days		8 (6–13)	—	
Disease‐modulating pharmacotherapy, *n* (%)	Yes	120 (69)	587 (47.1)	<0.001
	No	54 (31)	659 (52.9)	
	NSAID	5 (2.9)	81 (6.5)	
	Hydroxychloroquine	5 (2.9)	25 (2.0)	
	Favipiravir	18 (10.3)	175 (14.0)	
	Methylprednisolone	23 (13.2)	121 (9.7)	
	Clexane	37 (21.3)	273 (21.9)	
	Tocilizumab	20 (11.5)	110 (8.8)	
	Dexamethasone	100 (57.5)	455 (36.5)	
	Baricitinib	3 (1.7)	5 (0.4)	
Comorbidities, *n* (%)	Chronic liver disease	108 (62.1)	146 (11.7)	
	Metabolic dysfunction‐associated fatty liver disease (MAFLD)	99 (56.9)	126 (10.1)	
	Diabetes mellitus (DM)	64 (36.8)	388 (31.1)	
	Hypertension (HTN)	62 (35.6)	441 (35.4)	
	CKD, CCF, CLD	28 (16.1)	251 (20.1)	
	Cancer + IS	5 (2.9)	51 (4.1)	
Alcohol intake, *n* (%)	Yes	4 (2.3)	—	
	No	170 (97.7)		
Pattern of ALB, *n* (%)	Hepatocellular	42 (24.1)	205 (16.5)	
	Cholestasis	14 (8.1)	80 (6.4)	0.65
	Mixed	118 (67.8)	446 (35.8)	0.20
Prevalence of subtype of ALB, *n* (%)	Total bilirubin	33 (21.4)	153 (21.2)	
	ALP	39 (20.3)	135 (18.8)	
	ALT	130 (67.7)	475 (65.1)	
	AST	145 (75.5)	583 (82.3)	
	GGT	159 (82.8)	391 (54.4)	
Liver injury, *n* (%)	Yes	96 (55.2)	332 (26.6)	<0.001
	No	78 (44.8)	914 (73.4)	
Type of liver injury, *n* (%)	Hepatocellular	17 (17.7)	54 (4.3)	
	Cholestasis	31 (32.3)	149 (12.0)	0.23
	Mixed	48 (50)	123 (9.9)	0.52
Liver failure, *n* (%)	Yes	1 (0.6)	14 (1.1)	0.57
	No	173 (99.4)	1232 (98.9)	
ICU admission, *n* (%)	Yes	31 (17.8)	172 (13.8)	0.16
	No	143 (82.2)	1074 (86.2)	
Ventilator, *n* (%)	Yes	58 (33.3)	354 (28.4)	0.18
	No	116 (66.7)	892 (71.6)	
RRT, *n* (%)	Yes	2 (1.1)	—	
	No	172 (98.9)		
Inotrope, *n* (%)	Yes	1 (0.6)	—	
	No	173 (99.4)		
Death, *n* (%)	Yes	0 (0)	61 (4.9)	
	No	174 (100)	1185 (95.1)	

Nearly half of the (48.9%) patients had severe COVID‐19, while 31.8% and 18.8% had mild and moderate COVID‐19 infection, respectively. The commonest pharmacotherapy agents used were dexamethasone (57.5%), enoxaparin (21.3%), methylprednisolone (13.2%), and tocilizumab (11.5%). More than half of the patients had CLD (62.1%) and metabolic‐associated fatty liver disease (MAFLD) (56.9%), while 38% and 36.5% of the patients had type 2 diabetes mellitus and hypertension, respectively. Only 2.3% of patients in our cohort reported significant alcohol intake. The clinical outcomes of COVID‐19 in our patients, up to 6‐month duration, were as follows: 121 (69.5%) patients had complete resolution of illness, 39 (22.4%) patients developed “long COVID” sequelae, and 14 patients had missing data. Among the 39 patients with post‐COVID‐19 conditions, 63.7% had dyspnea on exertion, 22.7% had prolonged cough, and 13.6% had memory impairment.

The most common liver biochemistry derangement observed was of mixed pattern (64.6%), and most of the patients had abnormal GGT (82.8%), AST (75.5%), and ALT (67.7%). Half of the patients in our cohort had liver injury, and the commonest was again of mixed pattern (50%). Acute liver failure occurred only in one patient. The rate of ICU admission was 17.8%, and 33.3% patients needed ventilatory support.

There were no significant differences observed between the study cohort and the baseline cohort in the distribution of age, ethnicity, ALB, type of liver injury, rates of ICU admission, and rates of ventilator usage. There was a higher male gender preponderance, higher proportion of moderate and severe COVID‐19, higher proportion of the delta variant, higher use of disease‐modulating drugs, and higher rates of liver injury and liver failure in the study cohort as compared to the baseline cohort.

All patients underwent both ultrasound of abdomen and FibroScan; the rate of hepatic steatosis was 43.1% on ultrasound and 75.9% on FibroScan. Three patients (1.7%) had features of liver cirrhosis on ultrasound, while seven patients (3.4%) had elevated LSM suggestive of liver cirrhosis on FibroScan, and 16 patients (9.2%) had liver fibrosis (Table 2).

**Table 2:** Ultrasound abdomen and FibroScan results for COVID‐19 patients (*n* = 174)

Assessment		Number of patients
Abdomen ultrasound, *n* (%)	Normal	62 (35.6%)
	Fatty liver	75 (43.1%)
	Cirrhosis	3 (1.7%)
	Others	20 (11.5%)
	Missing	14 (8.0%)
FibroScan		
Liver stiffness Measurement	Mean (±SD), kPa	7.5 (± 5.0)
	Median (IQR), kPa	6.6 (5.1–8.3)
	Normal, *n* (%)	152 (87.4%)
	Liver fibrosis, *n* (%)	16 (9.2%)
	Liver cirrhosis, *n* (%)	6 (3.4%)
Continuous attenuation parameter	Mean (±SD), dB/m	288.3 (± 58.3)
	Median (IQR), dB/m	298.0 (245.8–333.8)
	S0, *n* (%)	42 (24.1%)
	S1, *n* (%)	17 (9.8%)
	S2, *n* (%)	13 (7.5%)
	S3, *n* (%)	102 (58.6%)

**Figure 2:**
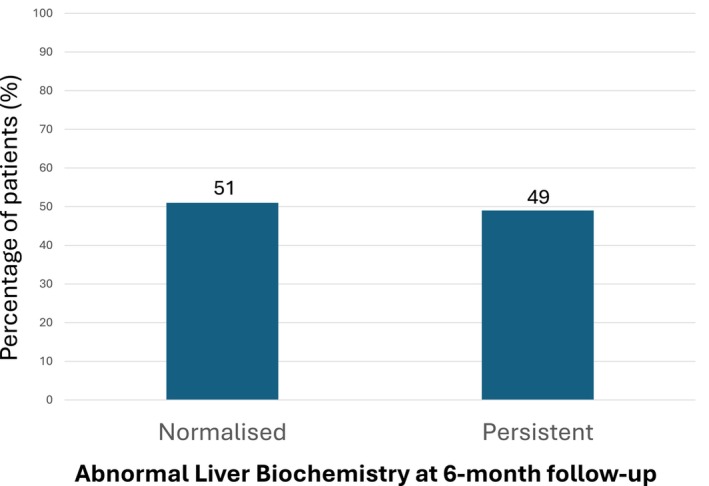
Proportion of patients with normalized and persistently abnormal liver biochemistry.

Approximately 52.3% of the patients had normalization of liver biochemistry recorded in the 6 months post discharge (Fig. 2). Comparing patients with persistent ALB and patients with normalized liver biochemistry, those with ALB had significantly higher mean BMI, higher mean serum LDL, higher rates of MAFLD and chronic liver disease, higher mean LSM and CAP score on FibroScan, and higher rates of moderate and severe COVID‐19.

On multivariate analysis, no statistically significant association was found (see Table 3).

**Table 3:** Predictive factors of persistent abnormal liver function post COVID‐19 infection (*n* = 174)

Factors	Normalized liver function post COVID		Univariate analysis, *P*‐value	Multivariate analysis, *P*‐value
	Yes, *n* = 91	No, *n* = 83		
Age				
Mean (± SD)	48.46 (± 14.94)	47.06 (± 14.35)	0.53	
≤60 years, *n* (%)	68 (41.8)	64 (58.2)	0.73	
>60 years, *n* (%)	23 (57.0)	19 (43.0)		
Gender *n*, (%)			0.50	
Male	55 (46.8)	46 (53.2)		
Female	36 (56.8)	37 (43.2)		
Ethnicity *n*, (%)			**0.01**	
Malay	45 (46.9)	57 (53.1)		0.38
Chinese	25 (64.3)	12 (35.7)		0.20
Indian	16 (43.8)	14 (56.3)		0.56
Others	5 (80)	0 (20)		0.31
BMI, mean (±SD)	28.47 (±5.85)	31.21 (±4.7)	**0.004**	0.47
Type of abnormal liver function, *n* (%)			0.82	
Hepatocellular	24 (57.8)	18 (42.2)		
Cholestasis	7 (78.3)	7 (21.7)		
Mixed	60 (43.5)	58 (56.5)		
Blood results				
FBS, (±SD)	8.2 (±3.0)	9.3 (±5.5)	0.57	
HbA1c (±SD)	8.2 (±2.7)	7.1 (±2.0)	0.67	
LDL (±SD)	3.0 (±0.7)	3.9 (±0.7)	**0.004**	0.9
TG (±SD)	1.8 (±1.0)	1.7 (±0.6)	0.92	
Comorbidities *n*, (%)				
DM	31 (50.7)	33 (49.3)	0.53	
MAFLD	43 (42.9)	56 (57.1)	**0.01**	0.08
Hypertension	29 (47.1)	33 (52.9)	0.34	
Chronic liver disease	48 (88.5)	60 (11.5)	**0.01**	0.45
Others	15 (39.3)	13 (60.7)	0.82	
Treatment, *n* (%)	68 (50.8)	42 (49.2)	0.10	0.83
Methylprednisolone	16 (57.1)	7 (42.9)	0.11	0.58
Favipiravir	10 (61.1)	8 (38.9)	0.80	
Tocilizumab	14 (41.7)	6 (58.3)	0.10	0.13
Dexamethasone	62 (49.1)	38 (50.9)	**0.004**	0.60
Clexane	20 (43.5)	17 (56.5)	0.85	
FibroScan TE				
Mean (±SD), kPa	6.6 (±3.5)	8.5 (±6.2)	**0.012**	0.125
Normal, *n* (%)	84 (51.8)	68 (48.2)	0.08	0.27
Liver fibrosis	6 (52.9)	10 (47.1)		0.48
Liver cirrhosis	1 (28.6)	5 (71.4)		0.13
FibroScan CAP				
Mean (±SD), dB/m	269.6 (±60.2)	308.8 (±48.7)	**<0.001**	0.8
S0, *n* (%)	30 (66.7)	12 (33.3)	**0.016**	0.49
S1	11 (57.1)	6 (42.9)		0.95
S2	6 (37.5)	7 (62.5)		0.2
S3	44 (45.5)	57 (54.5)		0.1
Liver injury, *n* (%)				
Yes	50 (42.7)	46 (57.3)	0.95	
Hepatocellular	4 (23.5)	13 (76.5)	0.078	0.12
Cholestasis	19 (51.6)	12 (48.4)		0.23
Mixed	27 (43.8)	21 (56.2)		0.45
Virus variant, *n* (%)			**0.047**	0.93
Cohort 1/pre‐DV	32 (35.2%)	42 (50.6%)		
Cohort 2 /DV	59 (64.8%)	41 (49.4%)		
COVID‐19 severity, *n* (%)			**0.015**	
Mild	23 (55.7)	31 (44.3)		0.24
Moderate	14 (44.4)	21 (55.6)		0.52
Severe	54 (50.5)	31 (49.5)		0.10
Ventilatory support, *n* (%)			0.63	
Yes	32 (44.3)	26 (55.7)		
ICU admission, *n* (%)			0.074	0.39
Yes	21 (54.5)	10 (45.5)		

Comparing patients with and without CLD (Table 4), a significant proportion of those with CLD were <60 years of age (65.9% *vs* 34.1%). Patients with CLD had higher BMI, lower ICU admission rate, and higher rate of normalization of LFT. Of note, no decompensation or clinical sequelae of portal hypertension were observed.

**Table 4:** Comparison of demographic and clinical characteristics of patients with and without underlying CLD (*n* = 174)

Factors	Underlying chronic liver disease		Univariate analysis, *P*‐value
	Yes, *n* = 108	No, *n* = 66	
Age			
Mean (±SD)	46.84 (±14.08)	49.35 (±15.48)	0.27
≤60 years, *n* (%)	87 (65.9)	45 (34.1)	0.07
>60 years, *n* (%)	21 (50.0)	21 (50.0)	
Gender, *n* (%)			0.53
Male	65 (64.4)	36 (35.6)	
Female	43 (58.9)	30 (41.1)	
Ethnicity, *n* (%)			0.11
Malay	66 (64.7)	36 (35.3)	
Chinese	18 (48.6)	19 (51.4)	
Indian	22 (73.3)	8 (26.7)	
Others	2 (40.0)	3 (60.0)	
BMI, mean (±SD)	30.97 (±5.24)	27.96 (±5.36)	**0.002**
Type of abnormal liver function, *n* (%)			0.06
Hepatocellular	30 (71.4)	12 (28.6)	
Cholestasis	5 (35.7)	9 (64.3)	
Mixed	73 (61.9)	45 (38.1)	
Blood results			
FBS (±SD)	8.6 (±4.7)	8.2 (±3.0)	0.49
HbA1c (±SD)	7.7 (±2.0)	7.5 (±2.8)	0.07
LDL (±SD)	3.4 (±0.9)	3.3 (±0.9)	0.70
TG (±SD)	2.0 (±1.0)	1.5 (±0.6)	0.22
Comorbidities, *n* (%)			
DM	45 (70.3)	19 (29.7)	0.11
Hypertension	41 (66.1)	21 (33.9)	0.42
Others	20 (71.4)	8 (30.6)	0.3
Treatment, *n* (%)	73 (62.4)	47 (37.6)	0.74
Methylprednisolone	11 (47.8)	12 (52.2)	0.17
Favipiravir	7 (38.9)	11 (61.1)	**0.041**
Tocilizumab	12 (60.0)	8 (40.0)	1.0
Dexamethasone	64 (64.0)	36 (36.0)	0.64
Clexane	21 (56.8)	16 (43.2)	0.57
FibroScan TE			
Mean (±SD), kPa	8.5 (±6.0)	6.0 (±2.2)	**<0.001**
Normal, *n* (%)	89 (58.6)	63 (41.4)	**0.027**
Liver fibrosis	13 (81.3)	3 (18.7)	
Liver cirrhosis	6 (100)	0 (0)	
Fibroscan CAP			
Mean (±SD), dB/m	307.0 (±50.2)	257.8 (±57.9)	**<0.001**
S0, *n* (%)	13 (31.0)	29 (69.0)	**<0.001**
S1	7 (41.2)	10 (59.8)	
S2	7 (53.8)	6 (46.2)	
S3	80 (79.2)	21 (20.8)	
Liver injury, *n* (%)			
Yes	58 (60.4)	38 (39.6)	0.64
Hepatocellular	14 (82.4)	3 (17.6)	0.21
Cholestasis	17 (54.8)	14 (45.2)	
Mixed	27 (56.3)	21 (43.7)	
Virus variant, *n* (%)			0.88
Cohort 1/pre‐DV	45 (60.8)	29 (39.2)	
Cohort 2/DV	63 (57.3)	37 (42.7)	
COVID‐19 severity, *n* (%)			0.33
Mild	30 (55.6)	24 (44.4)	
Moderate	25 (71.4)	10 (28.6)	
Severe	53 (94.6)	3 (5.4)	
Ventilatory support, *n* (%)			0.07
Yes	30 (51.7)	28 (48.3)	
ICU admission, *n* (%)			**0.004**
Yes	12 (38.7)	19 (61.3)	
Normalized LFT, *n* (%)			**0.012**
Yes	48 (52.7)	43 (47.3)	
No	60 (72.3)	23 (27.3)	

## Discussion

In our cohort of 174 patients, there was a high rate of severe COVID‐19 and use of disease‐modulating drugs. The commonest liver biochemistry derangement in our cohort was of a mixed pattern, and slightly over half of the patients were observed to have normalization of liver blood test during the follow‐up period. Hepatocellular type of liver injury was associated with persistently elevated liver biochemistry, while the converse was observed for Chinese ethnicity.

The commonest liver test parameter that was deranged in our cohort was GGT (82.8%), followed by AST (75.5%) and ALT (67.7%). There could be several reasons for the high rates of elevated GGT, which was not replicated in other studies that typically reported AST and ALT as the commonest parameters that were deranged. Partly it could be due to selection bias, as only 23.8% (*n* = 174) from the initial cohort of 713 patients with ALB were included in the final analysis. There was also notable age differences between our cohort with a median age of 50 years, as opposed to the mean age of >60 years for similar cohorts of patients reported in other studies.^3^, ^12^, ^15^ There can also be a marked difference in the severity as exemplified by a study of 102 patients from Singapore who had mostly mild COVID‐19 infection and a much younger mean age of 36 years, due to which there was predominantly raised transaminases and less than 4% elevated ALP.^16^

Furthermore, there was underreporting of cholestatic enzymes in the literature as indicated by a meta‐analysis of 56 studies comparing liver chemistry between severe and non‐severe COVID‐19 patients, which found that only 10 studies reported ALP values while only 6 reported GGT levels; while in another meta‐analysis of 107 studies, ALP and GGT were reported only in 5 and 6 studies, respectively.^6^, ^8^ Some studies have also reported hypoalbuminemia as the commonest liver test derangement.^11^, ^17^ We have opted against reporting albumin levels, as it may not accurately reflect liver function in the setting of an acute inflammatory state.^18^ The marked heterogeneity in the reporting of liver biochemistry across the numerous studies makes it challenging to make a fair comparison with our data.

Liver biochemistry returned to normal in approximately half of the patients in our cohort within the 6‐month follow‐up period. In a single‐center prospective study of 461 patients with COVID‐19 from China, there were 158 patients with elevated liver enzymes at discharge, and LFTs became normal in 73.4% of patients at 6 months follow‐up. The rate of normalization increased to 81% at 12 months.^14^ The rate of normalization of LFTs at 6 months was much higher than in our cohort, which could be explained by the younger median age (45 years) and lower proportion of severe COVID‐19 (20%) in addition to overreporting of patients with abnormal LFTs due to the inclusion of albumin as one of the parameters. In a large retrospective study from the United States, the rate of recovery of LFTs was 81.2% at a median follow‐up of 63 days among 692 patients. The ICU admission rate (15.6%) and need for invasive mechanical support (0.43%) were much lower than in our cohort, indicating a smaller proportion of patients with severe COVID‐19, which might explain the high rate of recovery.^19^ In a subset of 53 patients with follow‐up data post discharge in a retrospective study from Italy involving 161 patients with ALB, a trend towards normalization of liver biochemistry was observed at a median period of 36 days.^20^ In another study from China where follow‐up data for up to 2 months was available for 46 patients, a similar trend of normalization of LFTs was reported.^21^ One study with a follow‐up duration of 2 years for 149 patients reported that 15% had ALB; 17% previously had severe disease, but baseline data of LFTs were not reported.^22^ Overall, most studies report a trend towards high rates of normalization of liver biochemistry post discharge, which is concordant with our observation.

The commonest derangement in our cohort was of mixed pattern (64.6%). This was similar to what was reported in a multicenter Chinese study of 429 patients with liver impairment where 49% had mixed pattern, 39.6% had cholestatic pattern, and 11.1% had hepatocellular pattern.^15^ In our cohort, we found that patients with elevated ALT were more likely to have persistently deranged LFT whereas those with cholestasis showed higher proportion of LFT normalization. Patients who did not achieve normalization of LFTs also had significantly higher BMI and higher LDL level, and we postulate that the persistent deranged LFTs among patients with elevated ALT could be in part due to the presence of steatohepatitis driven by metabolic risk factors.

The use of disease‐modulating medications in our cohort was high at 68.8% and mirrors the combined prevalence of moderate and severe COVID‐19 infection, which is 68.3%. Drug‐induced liver injury is an important cause for deranged liver biochemistry as observed in a study among 1040 patients from Hong Kong, whereby the serum ALT and bilirubin were more likely to increase after the initiation of anti‐viral therapy and other immunomodulators.^10^ A meta‐analysis by Yadav *et al*. also found that patients with liver injury had considerably more use of lopinavir/ritonavir than those without liver injury.^9^ Interestingly, use of these medications did not affect or delay the normalization of LFT in our cohort, based on multivariate analysis.

High rates of hepatic steatosis on both ultrasound and FibroScan were observed in our cohort. This finding is in keeping with the high incidence of MAFLD in our general population (between 20% and 40%).^23^ Although our findings showed that patients with S0 and S1 steatosis were more likely to achieve normalization of LFT compared to those with S2 and S3 steatosis, this, however, did not reach statistical significance in multivariate analysis. This is much higher that the prevalence of steatosis as seen on computed tomography scans among 289 patients with moderate to severe COVID‐19 infection in Romania, which was 39.5%.^24^ The prevalence of CLD from two systematic reviews was around 4%^8^, ^9^, while another study reported it as 2.6%.^17^ These are much lower than the 12.5% observed in our cohort for elevated LSM, indicative of liver fibrosis including liver cirrhosis. The studies that the meta‐analyses were based on were mostly cross‐sectional and retrospective in nature with significant heterogeneity leading to possible under‐recognition of CLD. The use of different criteria for the detection of CLD might also have led to the variations observed, as exemplified by the report that severe fibrosis was present in 29.5% based on Fib‐4 values; which increased to 42.2% upon adjustment of the score for age >65 years among 289 patients from Romania.^24^ The rate of cirrhosis was low, but there was discordance between ultrasound and FibroScan (1.6% *vs* 3.6%). While ultrasound may not be accurate to detect early fibrosis, FibroScan can misdiagnose liver fibrosis in the setting of active inflammation and lead to overdiagnosis.^25^, ^26^

### Limitations

There are several limitations to our study. As previously mentioned, there may be selection bias, as only 26.3% of all patients with ALB met the inclusion criteria. Moreover, we could only study the association between COVID‐19 and ALB but not attribute causality, and further studies are needed to corroborate the pathogenic mechanism. The majority of CLD patients in this study had MAFLD, which may not be representative of other populations. However, MAFLD has been shown to be the most common CLD in our population.^27^

### Strengths

This study had the largest sample size reporting on the utility of transient elastography in the assessment of liver dysfunction post COVID‐19 infection to date.^28^, ^29^, ^30^ We were also able to report on the rate of normalization of LFTs within a 6‐month period, which is a longer follow‐up duration compared with other studies.

## Conclusion

In conclusion, in our cohort of COVID‐19 patients with liver function derangement, the commonest liver enzymes that were deranged were GGT, AST, and ALT. The liver dysfunction resolved in 52.3% of the patients during the 6‐month follow‐up period.

Persistently abnormal LFTs were associated with raised BMI, elevated serum LDL, increased rates of MAFLD and CLD, and higher rates of liver injury on univariate analysis but not on multivariate analysis.

## Data Availability

Data supporting the findings of this study are available within the article itself.
